# Supplementary material for: Work extraction from quantum systems with bounded fluctuations in work
Source: Nat Commun. 2016 Nov 25;7:13511. doi: 10.1038/ncomms13511 (PMC5133619; doi:10.1038/ncomms13511)
Supplement: Supplementary Information — Supplementary Notes 1-6 and Supplementary References. [file ncomms13511-s1.pdf]

I. SUPPLEMENTARY NOTE 1

A. Thermal operations with fluctuating work

In this supplementary note we introduce the framework that we will use to derive $W^{(c)}(\rho)$. The work extraction protocol is performed using a system ρ, a infinite thermal bath and a work system or weight.

First, let us characterize the type of process/operation that we consider, which we refer to as thermal operations with fluctuating work. Our setting consists of a system with Hamiltonian $H_{\rm S}$, a bath with Hamiltonian $H_{\rm B}$ initially in the thermal state $\rho_{\rm B} = \frac{1}{Z_{\rm B}} {\rm e}^{-\beta H_{\rm B}}$, and an ideal weight with Hamiltonian $H_{\rm W} = \int_{\mathbb{R}} dx\, x |x\rangle\langle x|$, where the orthonormal basis $\{|x\rangle, \forall\, x \in \mathbb{R}\}$ represents the position of the weight. Any joint transformation of system, bath and weight is represented by a Completely Positive Trace Preserving (CPTP) map $\Gamma_{\rm SBW}$ satisfying the following conditions:

Microscopic reversibility (Second Law): It has an (CPTP) inverse $\Gamma_{\rm SBW}^{-1}$, which implies unitarity $\Gamma_{\rm SBW}(\rho_{\rm SBW}) = U \rho_{\rm SBW} U^\dagger$.

Energy conservation (First Law): $[U, H_{\rm S} + H_{\rm B} + H_{\rm W}] = 0$.

Independence from the "position" of the weight: The unitary commutes with the translations on the weight $[U, \Delta_{\rm W}] = 0$. The generator of the translations $\Delta_{\rm W}$ is canonically conjugated to the position of the weight $[H_{\rm W}, \Delta_{\rm W}] = {\rm i}$.

Classicality of work: Before and after applying the global map $\Gamma_{\rm SBW}$ the position of the weight is measured, obtaining outcomes $|x\rangle$ and $|x + w\rangle$ respectively. The joint transformation of the system and work random variable w is given by the map

$$\Lambda(\rho_{\rm S}, w) = \int_{\mathbb{R}} dx\, {\rm tr}_{\rm BW} \left[Q_{x+w}\, U \left(\rho_{\rm S}\, \rho_{\rm B}\, Q_x \rho_{\rm W} Q_x \right) U^\dagger \right] \ , \tag{1}$$

where $Q_x = |x\rangle\langle x|$ is a weight eigen-projector.

Condition $[U, \Delta_{\rm W}] = 0$ implies that the reduced map on system and bath is a mixture of unitaries (Theorem 1 in [1]). Hence, this transformation can never decrease the entropy of system and bath, which guarantees that the weight is not used as a source of non-equilibrium. Note that after integrating over the work varable

$$\int_{\mathbb{R}} dw\, \Lambda(\rho_{\rm S}, w) = {\rm tr}_{\rm BW} \left[U \left(\rho_{\rm S}\, \rho_{\rm B}\, \Theta[\rho_{\rm W}] \right) U^\dagger \right] = \Gamma_{\rm S}(\rho_{\rm S}) \ , \tag{2}$$

we obtain the reduced CPTP map for the system. The state $\Theta[\rho_{\rm W}] = \int_{\mathbb{R}} dx\, Q_x \rho_{\rm W} Q_x$ is the energy-diagonal version of $\rho_{\rm W}$. But $\Lambda(\rho_{\rm S}, w)$ being independent of $\rho_{\rm W}$, we can choose the initial state to be diagonal $\Theta[\rho_{\rm W}]$. Also, note that when tracing the system

$$ {\rm tr}_{\rm S} \Lambda(\rho_{\rm S}, w) = P(w) \ , \tag{3}$$

we obtain the probability distribution of the work generated in the transformation.

B. Thermal operations with non-constant Hamiltonian

Thermal operations are general enough to include the case where the initial Hamiltonian of the system $H_{\rm S}$ is different than the final one $H'_{\rm S}$. This is done by including an additional qubit X which plays the role of a switch (as in [2, 3]). Now the total Hamiltonian is

$$H = H_{\rm S} \otimes |0\rangle_{\rm X}\langle 0| + H'_{\rm S} \otimes |1\rangle_{\rm X}\langle 1| + H_{\rm B} + H_{\rm W} \ , \tag{4}$$

and energy conservation reads $[V, H] = 0$, where V is the global unitary when we include the switch. We impose that the initial state of switch is $|0\rangle_{\rm X}$ and the global unitary V performs the switching

$$V \left(\rho_{\rm SBW} \otimes |0\rangle_{\rm X}\langle 0| \right) V^\dagger = \rho'_{\rm SBW} \otimes |1\rangle_{\rm X}\langle 1| \ , \tag{5}$$

for any $\rho_{\rm SBW}$. This implies

$$V = U \otimes |1\rangle_{\rm X}\langle 0| + \tilde{U} \otimes |0\rangle_{\rm X}\langle 1| \ , \tag{6}$$

where U and $\tilde{U}$ are unitaries on system, bath and weight. Condition $[V, H] = 0$ implies

$$U(H_{\rm S} + H_{\rm B} + H_{\rm W}) = (H'_{\rm S} + H_{\rm B} + H_{\rm W})U \ . \tag{7}$$

Therefore, the reduced map on system, bath and weight can be written as

$$\Gamma_{\rm SBW}(\rho_{\rm SBW}) = U \rho_{\rm SBW} U^\dagger \ , \tag{8}$$

where the unitary U does not necessarily commute with $H_{\rm S} + H_{\rm B} + H_{\rm W}$ nor $H'_{\rm S} + H_{\rm B} + H_{\rm W}$ but satisfies (7).

C. Reducing the quantum problem to the classical case

Let us show that the equation connecting the initial state of the system $\rho_{\rm S}$ with the final one conditioned on work w,

$$\rho'_{{\rm S}|w} = \frac{1}{P(w)} \Lambda(\rho_{\rm S}, w) \ , \tag{9}$$

decouples in the diagonal part and the other energy modes [4–6]. The map Θ_α defined as

$$\Theta_\alpha[\rho_{\rm S}] = \int_{\mathbb{R}} dt\, {\rm e}^{i\alpha t}\, {\rm e}^{iH_{\rm S}t} \rho_{\rm S} {\rm e}^{-iH_{\rm S}t} \ , \tag{10}$$

projects the state $\rho_{\rm S}$ onto the α-energy mode of $H_{\rm S}$. When $\alpha = 0$ it projects the state onto its diagonal, when written in the energy eigenbasis. And in general, it projects the state onto all the terms $|s_1\rangle\langle s_2|$ such that $\mathcal{E}_{s_1} - \mathcal{E}_{s_2} = \alpha$. Using constraint (7) and identities ${\rm e}^{itH_{\rm W}} Q_x = {\rm e}^{itx}$ and $[H_{\rm B}, \rho_{\rm B}] = 0$ we obtain

$$\begin{aligned}
\Theta_\alpha[\Lambda(\rho_{\rm S}, w)] &= \int_{\mathbb{R}} dt\, {\rm e}^{i\alpha t}\, {\rm e}^{iH'_{\rm S}t}\, \Lambda(\rho_{\rm S}, w)\, {\rm e}^{-iH'_{\rm S}t} \\
&= \int_{\mathbb{R}} dt\, dx\, {\rm e}^{i\alpha t}\, {\rm tr}_{\rm BW} \left[{\rm e}^{i(H'_{\rm S}+H_{\rm B}+H_{\rm W})t} Q_{x+w}\, U \left(\rho_{\rm S}\, \rho_{\rm B}\, Q_x \rho_{\rm W} Q_x\right) U^\dagger {\rm e}^{-i(H'_{\rm S}+H_{\rm B}+H_{\rm W})t} \right] \\
&= \int_{\mathbb{R}} dt\, dx\, {\rm e}^{i\alpha t}\, {\rm tr}_{\rm BW} \left[Q_{x+w}\, U \left({\rm e}^{i(H_{\rm S}+H_{\rm B}+H_{\rm W})t} \rho_{\rm S}\, \rho_{\rm B}\, Q_x \rho_{\rm W} Q_x {\rm e}^{-i(H_{\rm S}+H_{\rm B}+H_{\rm W})t} \right) U^\dagger \right] \\
&= \int_{\mathbb{R}} dt\, dx\, {\rm e}^{i\alpha t}\, {\rm tr}_{\rm BW} \left[Q_{x+w}\, U \left({\rm e}^{iH_{\rm S}t} \rho_{\rm S} {\rm e}^{-iH_{\rm S}t}\, \rho_{\rm B}\, Q_x \rho_{\rm W} Q_x \right) U^\dagger \right] \\
&= \Gamma_{\rm S}(\Theta_\alpha[\rho_{\rm S}], w) \ ,
\end{aligned} \tag{11}$$

as claimed above. This shows that when the initial state is diagonal, $\Theta_\alpha[\rho_{\rm S}] = 0$ for all $\alpha \neq 0$, so is the final one; and visa-versa. Thus our results, concerning work extraction and state formation where either the initial or final state diagonal in the energy eigenbasis, are valid in the case that the non-equilibrium state involves coherences between energy eigenstates.

The diagonal part of the map is nicely characterized by the conditional probability distribution

$$t(s',\, w|\, s) = \langle s'|\Lambda(|s\rangle\langle s|, w)|s'\rangle \ , \tag{12}$$

where $|s\rangle$ is the eigenbasis of $\Theta[\rho_{\rm S}]$ and $|s'\rangle$ is the eigenbasis of $\Theta[\Lambda(\rho_{\rm S}, w)]$. Note that the "dynamics" of the diagonals is a completely classical problem. From now on we use $\rho = \Theta[\rho_{\rm S}]$, $\rho' = \Gamma_{\rm S}\Theta[\rho_{\rm S}]$ for the initial/final states in optimal work extraction / state formation processes.

D. Necessary condition for thermal operations

Using (7) and definitions made above we obtain

$$\begin{aligned}
&\sum_s \int_{\mathbb{R}} dw\, t(s', w|s)\, {\rm e}^{\beta(\mathcal{E}_{s'} - \mathcal{E}_s + w)} \\
&= \sum_s \int_{\mathbb{R}} dw\, dx\, \langle s'|\, {\rm e}^{\beta H'_{\rm S}}\, {\rm tr}_{\rm BW} \left[{\rm e}^{\beta H_{\rm W}} Q_{x+w}\, U \left({\rm e}^{-\beta H_{\rm S}} |s\rangle\langle s|\, \rho_{\rm B}\, {\rm e}^{-\beta H_{\rm W}} Q_x \rho_{\rm W} Q_x \right) U^\dagger \right] |s'\rangle \\
&= \int_{\mathbb{R}} dx'\, dx\, \langle s'|\, {\rm e}^{\beta H'_{\rm S}}\, {\rm tr}_{\rm BW} \left[{\rm e}^{\beta H_{\rm W}} Q_{x'}\, U \left({\rm e}^{-\beta H_{\rm S}} \frac{{\rm e}^{-\beta H_{\rm B}}}{Z_{\rm B}} {\rm e}^{-\beta H_{\rm W}} Q_x \rho_{\rm W} Q_x \right) U^\dagger \right] |s'\rangle \\
&= \langle s'|\, {\rm e}^{\beta H'_{\rm S}}\, {\rm tr}_{\rm BW} \left[{\rm e}^{\beta H_{\rm W}} {\rm e}^{-\beta H'_{\rm S}} \frac{{\rm e}^{-\beta H_{\rm B}}}{Z_{\rm B}} {\rm e}^{-\beta H_{\rm W}} U \Theta[\rho_{\rm W}] U^\dagger \right] |s'\rangle \\
&= {\rm tr}_{\rm SBW} \left[|s'\rangle\langle s'| \frac{{\rm e}^{-\beta H_{\rm B}}}{Z_{\rm B}} U \Theta[\rho_{\rm W}] U^\dagger \right] \ .
\end{aligned} \tag{13}$$

As mentioned above, Theorem 1 in [third law masanes oppenheim] proves that condition $[U, \Delta_{\rm W}] = 0$ implies

$$ {\rm trW} \left[U \mathbb{I}_{\rm SB} \rho_{\rm W} U^\dagger \right] = \mathbb{I}_{\rm SB} \tag{14}$$

for all $\rho_{\rm W}$. Applying this to (13) we obtain

$$\mathrm{tr}_{\rm SBW}\left[|s'\rangle\langle s'|\frac{\mathrm{e}^{-\beta H_{\rm B}}}{Z_{\rm B}}U\Theta[\rho_{\rm W}]U^\dagger\right] = \mathrm{tr}_{\rm SB}\left[|s'\rangle\langle s'|\frac{\mathrm{e}^{-\beta H_{\rm B}}}{Z_{\rm B}}\right] = 1\ . \tag{15}$$

This proves the "only if" part of the following

Theorem 1. The (classical) map $t(s',w|s)$ comes from a thermal operation

$$t(s',w|s) = \langle s'|\Lambda(|s\rangle\langle s|,w)|s'\rangle\ , \tag{16}$$

if and only if

$$\sum_s \int_{\mathbb{R}} dw\, t(s',w|s)\, \mathrm{e}^{\beta(\mathcal{E}_{s'}-\mathcal{E}_s+w)} = 1\ , \tag{17}$$

for all s'.

The "if" part of the above theorem is proven in Theorem 5 from [7].

E. Optimal thermal operations have minimal work fluctuations

Given a process $t(s',w|s)$, satisfying (17), the work generated in a particular state transition $s\to s'$ has probability distribution

$$t(w|s,s') = \frac{t(s',w|s)}{t(s'|s)}\ , \tag{18}$$

where $t(s'|s) = \int_{\mathbb{R}} dw\, t(s',w|s)$ is the value transformation on the system. In general, despite the conditioning on s,s', the distribution (18) contains fluctuations on w. In certain setups, a less fluctuating work variable w is desirable. The following theorem shows that this can always be done without decreasing the average work generated in the given process $t(s',w|s)$.

Theorem 2. If $t(s',w|s)$ satisfies condition (17) then

$$\tilde{t}(s',w|s) = \delta(w-w(s'|s))t(s'|s) \tag{19}$$

with

$$w(s'|s) = T\ln\int_{\mathbb{R}} dw\, t(w|s,s')\,\mathrm{e}^{\beta w} \tag{20}$$

also satisfies (17), and in addition

$$\langle w\rangle_{\tilde{t}} \;\geq\; \langle w\rangle_t\ , \tag{21}$$

$$\max_{w\,:\,\tilde{t}(w)>0} |w-\langle w\rangle_{\tilde{t}}| \;\leq\; \max_{w\,:\,t(w)>0} |w-\langle w\rangle_t|\ . \tag{22}$$

The above inequalities are saturated if and only if $\tilde{t}(w,s'|s) = t(w,s'|s)$.

To show that $\tilde{t}(s',w|s)$ satisfies (17), first, exponentiate the two sides of (20),

$$\mathrm{e}^{\beta w(s'|s)} = \int_{\mathbb{R}} dw\, t(w|s,s')\,\mathrm{e}^{\beta w}\ , \tag{23}$$

and second, multiply by $t(s'|s)\,\mathrm{e}^{\beta(\mathcal{E}_{s'}-\mathcal{E}_{\rm S})}$ and sum over s,

$$\sum_s t(s'|s)\,\mathrm{e}^{\beta(\mathcal{E}_{s'}-\mathcal{E}_{\rm S}+w(s'|s))} = \sum_s\int_{\mathbb{R}} dw\, t(s',w|s)\,\mathrm{e}^{\beta(\mathcal{E}_{s'}-\mathcal{E}_{\rm S}+w)} = 1\ . \tag{24}$$

Note that the two maps, $t(s',w|s)$ and $\tilde{t}(s',w|s)$, have the same value $t(s'|s)$. That is, they perform the same transformation on the system.

To show (21) we use the convexity of the exponential in equation (20), obtaining

$$w(s'|s) \geq T\ln\mathrm{e}^{\int_{\mathbb{R}} dw\, P(w|s,s')\,\beta\, w} = \int_{\mathbb{R}} dw\, t(w|s,s')\, w\ . \tag{25}$$

Averaging over s,s' gives (21). Also, note that due to strict convexity of the exponential, the equality in (25) is only achieved when $t(w|s,s') = \delta(w-w(s'|s)) = \tilde{t}(w|s,s')$.

To show (22), note that the convexity of the exponential implies that, unless $t(w|s,s') = \delta(w-w(s'|s))$, there are values $w(+) > w(s'|s)$ and $w(-) < w(s'|s)$ such that $t(w(\pm)|s,s') > 0$. Hence, equality is only achieved when $t=\tilde{t}$.

II. SUPPLEMENTARY NOTE 2

In this supplementary note we calculate the maximal average work extracted (or minimal work of formation) for the state transformation $\rho \to \rho'$ with unbounded fluctuations. The work is given by the difference in free energy. By explicitly calculating the work distribution we show that the work fluctuations diverge as the initial or final system states become more athermal. We also show that there is no map that can achieve the optimal average work (i.e. thermodynamically reversible) with a smaller range of fluctuations about the average (see also Supplementary Note 1E).

In the previous Supplementary Notes we simplified the work-optimal map to a form where it is defined by the map parameters $\{t(s'|s), w(s'|s)\}$ where $t(s'|s)$ defined the reduced map on the system. We have shown that, in the case that the initial or final state is diagonal we can work with de-phased initial and final states. Furthermore the $t(s'|s)$ must obey

$$1 = \sum_{s'} t(s'|s) \tag{26}$$

$$x_{s'} = \sum_{s} x_s t(s'|s) \tag{27}$$

$$1 = \sum_{s} t(s'|s)\, \mathrm{e}^{\beta(\mathcal{E}_{s'} - \mathcal{E}_s + w(s'|s))} \tag{28}$$

where the first two constraints ensure that the reduced map acting on the system is stochastic and achieves the desired state transformation, and the third constraint, derived in the previous section, is required to ensure that the map is microscopically reversible. In the following we simplify the map further in the case of optimal work extraction.

It will be useful to relax the reversibility equality to an inequality

$$1 \geq \sum_{s} t(s'|s)\, \mathrm{e}^{\beta(\mathcal{E}_{s'} - \mathcal{E}_s + w(s'|s))} \tag{29}$$

where saturation of the inequality implies that the map is an allowed (thermal) operation. In all future calculations we make use of this relaxed constraint and show that our solutions saturate the inequality.

The average work associated with the optimal map achieving $\rho \to \rho'$ is given by

$$W = \sum_{ss'} x_s t(s'|s) w(s'|s) \tag{30}$$

it turns out to be sufficient to optimize this under the reversibility constraint (29). The Lagrangian is

$$\mathcal{L} = \sum_{ss'} x_s t(s'|s) w(s'|s) + \sum_{s'} \lambda_{s'} \left(1 - \sum_{ss'} t(s'|s) e^{\beta(w(s'|s) + \Delta\mathcal{E}_{ss'})} \right) \tag{31}$$

Maximising with respect to $w(s'|s)$ gives

$$w(s'|s) = \frac{1}{\beta} \log \left(\frac{x_s e^{\beta \Delta\mathcal{E}_{ss'}}}{\lambda_{s'} \beta} \right) \tag{32}$$

Extremizing with respect to $\lambda_{s'}$ and applying (26) and (27) gives

$$\lambda_{s'} = \frac{x_{s'}}{\beta} \tag{33}$$

Substituting this into (32) gives the optimal work

$$\sum_{ss'} x_s t(s'|s) \frac{1}{\beta} \log \left(\frac{x_s e^{\beta \Delta\mathcal{E}_{ss'}}}{x_{s'}} \right) = F(\rho) - F(\rho') \tag{34}$$

where $F(\rho) = \langle \mathcal{E}(\rho) \rangle - 1/\beta\, S(\rho)$ is the standard free energy and we have again used (26), (27). Notice that the result is independent of our choice of $t(s'|s)$, i.e. any map that takes us from $\rho \to \rho'$ gives the same optimal work. This is simply a statement that the free energy is a state variable (i.e. is path independent). The work $W^{(\infty)}$ is the average of the work values, gives by

$$w(s'|s) = \frac{1}{\beta} \log \left(\frac{x_s e^{\beta \Delta\mathcal{E}_{ss'}}}{x_{s'}} \right) \tag{35}$$

Note work values with $x_s = 0$ are set to zero (they have zero probability of occurring in the work distribution). Note that the fluctuations diverge as the initial / final state moves further from equilibrium. It is easy to check that substituting our solutions for $w(s'|s)$ into (29) saturates the inequality, therefore this map is achievable with thermal operations.

Theorem 2 shows that no thermal map can achieve this W with smaller worst case fluctuations. Having explicitly calculated the work values, we have derived necessary and sufficient conditions for a thermal map to exist that achieves this W (i.e. the thermodynamically reversible work) given that the fluctuations are constrained $|w - W| \leq c$

Result 1. The process $(\rho,\, H_{\mathrm{S}}) \to (\rho',\, H'_{\mathrm{S}})$ can be achieved in a thermodynamically reversible map if

$$e^{\beta(\Delta F(\rho\to\rho')-c)} \leq \frac{x_s e^{\beta\mathcal{E}_s}}{x_{s'} e^{\beta\mathcal{E}_{s'}}} \leq e^{\beta(\Delta F(\rho\to\rho')+c)} \quad \forall\, s,\, s' \tag{36}$$

This becomes a necessary and sufficient condition if the initial and/or or final state is diagonal in the energy eigenbasis

Proof. Given Theorem 2, it is sufficient to find the conditions that the work values (35) obey the c-bound

$$\left| \frac{1}{\beta} \log\left(\frac{x_s e^{\beta\Delta\mathcal{E}_{ss'}}}{x_{s'}} \right) - \Delta F(\rho \to \rho') \right| \leq c \tag{37}$$

which gives the desired inequalities □

III. SUPPLEMENTARY NOTE 3

In this Supplementary Note we derive the c-bounded work content for general quantum state ρ.

A. determining the optimal map

Lemma 1. We can always find an optimal protocol where $t(s'|s) \to \tilde{t}_s = e^{-\beta\mathcal{E}_{s'}}/\mathcal{Z}'$, where $\mathcal{Z}' = \mathrm{tr}[e^{-\beta H'_{\mathrm{S}}}]$, and $w(s'|s) \to \tilde{w}_s$ that obeys $|\tilde{w}(s) - \langle W\rangle| \leq |w(s'|s) - \langle W\rangle|\ \forall\ s, s'$

Proof. The average work extracted by a given protocol is given by

$$W = \sum_{ss'} x_s t(s'|s) w(s'|s) \tag{38}$$

where $\{t(s'|s), w(s'|s)\}$ obey constraints (26) (27) and (29).

Assume there exists some optimal protocol $\{t(s'|s), w(s'|s)\}$ that satisfies these and obeys the c-bound

$$|w(s'|s) - W| \leq c \quad \forall s,\, s' \tag{39}$$

Define a new protocol with

$$\tilde{t}(s') = \frac{e^{-\beta\mathcal{E}_{s'}}}{\mathcal{Z}'} \tag{40}$$

$$\tilde{w}(s) = \sum_k t(k|s) w(k|s) \tag{41}$$

The average work extracted by this protocol is

$$\tilde{W} = \sum_{ss'} x_s \tilde{t}(s')\tilde{w}(s) = \sum_s x_s \tilde{w}(s) = \sum_{ss'} x_s t(s'|s) w(s'|s) \tag{42}$$

therefore it extract the same amount of work as the optimal protocol. It is also obeys the microscopic reversibility

inequality (29) as

$$\begin{aligned}\sum_s \tilde{t}(s')e^{\beta(\tilde{w}(s)-\Delta\mathcal{E}_{ss'})} &= \frac{1}{\mathcal{Z}'}\sum_s e^{\beta\left(\sum_k t(k|s)w(k|s)-\mathcal{E}_s\right)}\\ &\leq \frac{1}{\mathcal{Z}'}\sum_{sk} t(k|s)e^{\beta w(k|s)-\beta\mathcal{E}_s}\\ &= \frac{1}{\mathcal{Z}'}\sum_{sk} t(k|s)e^{\beta w(k|s)-\beta\mathcal{E}_s+\beta\mathcal{E}_k-\beta\mathcal{E}_k}\\ &\leq \frac{1}{\mathcal{Z}'}\sum_k e^{-\beta\mathcal{E}_k}\\ &= 1\end{aligned}$$

where we have used the fact that $\sum_k t(k|s) = 1$ and the convexity of the exponential function to get $e^{\beta\tilde{w}(s)} \leq \sum_k t(k|s)e^{\beta w(k|s)}$, and in the second to last line have used the reversibility of the original map. Finally, the new protocol also obeys the c-bound as the work values $\tilde{w}(s)$ are convex sums of the work values $w(s'|s)$ and therefore $\max\{w(k|s)\} \geq \tilde{w}(s) \geq \min\{w(k|s)\}$. As W is unchanged then the worst case fluctuations of the new work distribution about the average must be less than or equal to those of the optimal distribution. □

We now drop the tilde from $\tilde{w}(s)$ and $\tilde{t}(s')$. It is simple to check that $t(s') = 1/\mathcal{Z}'e^{-\beta\mathcal{E}_{s'}}$ satisfies the conditions (26) and (27). The reversibility inequality (29) becomes

$$\mathcal{Z}' \geq \sum_s e^{\beta(w(s)-\mathcal{E}_s)} \tag{43}$$

and the average work is given by

$$W = \sum_s x_s w(s) \tag{44}$$

We are now in a position to derive $W^{(c)}(\rho)$. The Lagrangian is the same as employed in the previous section except that it includes terms bounding all $|w(s) - \sum_k x_k w(k)| \leq 0$. Due to the exponential in the reversibility term $\mathcal{Z}' \geq \sum_s e^{\beta(w(s)-\mathcal{E}_s)}$ we must linearise these bounds so as to avoid generating a transcendental equation when extremizing the Lagrangian over $w(s)$. We therefore select the bounds

$$w(s) - \sum_k x_k w(k) \;\leq\; c \tag{45}$$

$$\sum_k x_k w(k) - w(s) \;\leq\; c \tag{46}$$

with associate Lagrange parameters $\mu_{ss'}$ and $\bar{\mu}_{ss'}$. As all fluctuations are either positive ($w(s) \geq W^{(c)}(\rho)$) or negative ($w(s) < W^{(c)}(\rho)$) one of these bounds will always be trivial for each work value $w(s)$. Including the reversibility constraint (43) we maximise the average work $W = \sum_s x_s w(s)$ by extremizing the Lagrangian

$$\mathcal{L} = \sum_s f_s w(s) + \lambda\left(\mathcal{Z}' - \sum_s e^{\beta(w(s)-\mathcal{E}_s)}\right) + c\sum_s(\mu_s + \bar{\mu}_s) \tag{47}$$

where

$$f_s = x_s f - (\bar{\mu}_s - \mu_s) \tag{48}$$

where $f = 1 + \sum_k(\bar{\mu}_k - \mu_k)$ and we have replaced the old Lagrange parameters with $\lambda = \mathcal{Z}'\sum_s \lambda_s$ and $\mu_s(\bar{\mu}_s) = \sum_{s'} \mu_{ss'}(\bar{\mu}_{ss'})$. Maximizing w.r.t $w(i)$ and λ gives

$$w(s) \;=\; \frac{1}{\beta}\log\left(\frac{f_s e^{\beta\mathcal{E}_s}}{\beta\lambda}\right) \tag{49}$$

$$\lambda \;=\; \frac{1}{\beta\mathcal{Z}'} \tag{50}$$

where we have used $\sum_s f_s = 1$. Substituting these into the Lagrangian simplifies it to

$$\mathcal{L} = \frac{1}{\beta}\sum_s f_s \log\left(f_s e^{\beta\mathcal{E}_s}\mathcal{Z}'\right) + c\sum_s(\mu_s + \bar{\mu}_s) \tag{51}$$

Maximizing w.r.t μ_j and $\bar{\mu}_j$ under the condition $\mu_j \geq 0$ and $\bar{\mu}_j \geq 0$ gives

$$\frac{\partial \mathcal{L}}{\partial \mu_j} = 0 \;\rightarrow\; \log\left(f_j e^{\beta \mathcal{E}_j}\right) - \sum_s x_s \log\left(f_s e^{\beta \mathcal{E}_s}\right) = -\beta c \tag{52}$$

$$\frac{\partial \mathcal{L}}{\partial \bar{\mu}_j} = 0 \;\rightarrow\; -\log\left(f_j e^{\beta \mathcal{E}_j}\right) + \sum_s x_s \log\left(f_s e^{\beta \mathcal{E}_s}\right) = -\beta c \tag{53}$$

where we have used

$$\frac{\partial f_s}{\partial \mu_j} = -x_s + \delta_{sj} \tag{54}$$

$$\frac{\partial f_s}{\partial \bar{\mu}_j} = x_s - \delta_{sj} \tag{55}$$

For $c > 0$, equations (52) and (53) cannot be simultaneously satisfied by any $w(j) = 1/\beta \log\left(f_j e^{\beta \mathcal{E}_j} \mathcal{Z}'\right)$ therefore if $\mu_j > 0$ then $\bar{\mu}_j = 0$ and visa-versa. As $w(i) = 1/\beta \log\left(f_i e^{\beta \mathcal{E}_i} \mathcal{Z}'\right)$ equations (52) and (53) can be written in the form

$$\mu_j \neq 0 \rightarrow w(j) - \langle W \rangle = -c \tag{56}$$

$$\bar{\mu}_j \neq 0 \rightarrow w(j) - \langle W \rangle = c \tag{57}$$

Therefore fluctuations saturate either a positive or negative bound, or saturate no bounds ($\mu_j = \bar{\mu}_j = 0$). It will therefore be useful to partition the set of energy levels into those for which the resulting fluctuations will saturate a positive bound $i \in \mathcal{X}_+$, a negative bound $j \in \mathcal{X}_-$ or saturate no bounds $u \in \mathcal{X}_u$. For work values $w(i)$, $w(i')$ that saturate a positive bound and $w(j)$, $w(j')$ that saturate a negative bound we have

$$f_i e^{\beta \mathcal{E}_i} = f_{i'} e^{\beta \mathcal{E}_{i'}} \tag{58}$$

$$f_j e^{\beta \mathcal{E}_j} = f_{j'} e^{\beta \mathcal{E}_{j'}} \tag{59}$$

$$f_j e^{\beta \mathcal{E}_j} = f_{i'} e^{\beta (\mathcal{E}_{i'} - 2c)} \tag{60}$$

$$f_i = x_i f - \bar{\mu}_i \tag{61}$$

$$f_j = x_j f + \mu_j \tag{62}$$

$$f_u = x_u f \tag{63}$$

where (58) comes from summing (52) for μ_j, $\mu_{j'}$, (59) from summing (53) for $\bar{\mu}_i$, $\bar{\mu}_{i'}$, (60) comes from subtracting (52) for μ_j and (53) for $\bar{\mu}_i$ and (61)–(63) are just the definition (48) with the conditions $\bar{\mu}_j = 0$, $\mu_i = 0$ and $\mu_u = \bar{\mu}_u = 0$ applied. (58)–(60) and (63) allow us to simplify (52) and (53) to

$$\frac{\partial \mathcal{L}}{\partial \mu_j} = 0 \rightarrow \log\left(\frac{f}{f_j e^{\beta \mathcal{E}_j}}\right) = \nu + c \tag{64}$$

$$\frac{\partial \mathcal{L}}{\partial \bar{\mu}_i} = 0 \rightarrow \log\left(\frac{f}{f_i e^{\beta \mathcal{E}_i}}\right) = \nu - c \tag{65}$$

$$\nu = \frac{\beta}{X_u}\left(\frac{1}{\beta} H_u(\rho) + c(X_- - X_+) - \langle \mathcal{E}_u(\rho) \rangle\right) \tag{66}$$

Where $X_+ = \sum_{s \in \mathcal{X}_+} x_s$, $X_- = \sum_{s \in \mathcal{X}_-} x_s$, $X_u = \sum_{s \in \mathcal{X}_u} x_s$, $H_u(\rho) = -\sum_{s \in \mathcal{X}_u} x_s \log x_s$ and $\langle \mathcal{E}_u(\rho) \rangle = \sum_{s \in \mathcal{X}_u} x_s \mathcal{E}_s$. (58)–(63) let us to relate the remaining Lagrange parameters by

$$\bar{\mu}_{i'} = f(x_{i'} - x_i e^{\beta(\mathcal{E}_i - \mathcal{E}_{i'})}) + \bar{\mu}_i e^{\beta(\mathcal{E}_i - \mathcal{E}_{i'})} \tag{67}$$

$$\mu_{j'} = f(x_j e^{\beta(\mathcal{E}_j - \mathcal{E}_{j'})} - x_{j'}) + \mu_j e^{\beta(\mathcal{E}_j - \mathcal{E}_{j'})} \tag{68}$$

$$\bar{\mu}_i = \frac{(k - \mu_j)(e^{\beta \mathcal{E}_i} x_i - e^{\beta(2c - \mathcal{E}_j)} x_j) - \mu_j e^{\beta(2c - \mathcal{E}_j)}}{(1 - x_i) e^{\beta \mathcal{E}_i} + x_j e^{\beta(2c - \mathcal{E}_j)}} \tag{69}$$

where $k = f - \bar{\mu}_i + \mu_j$. Summing (67) over i' and (68) over j' gives

$$\sum_{i'} \bar{\mu}_{i'} = f(X_+ - x_i e^{\beta \mathcal{E}_i} \mathcal{Z}_+) + \bar{\mu}_i e^{\beta \mathcal{E}_i} \mathcal{Z}_+ \tag{70}$$

$$\sum_{j'} \mu_{j'} = f(x_j e^{\beta \mathcal{E}_j} \mathcal{Z}_- - X_i) + \mu_j e^{\beta \mathcal{E}_j} \mathcal{Z}_- \tag{71}$$

where $\mathcal{Z}_+ = \sum_{s\in\mathcal{X}_+} e^{-\beta\mathcal{E}_s}$ and $\mathcal{Z}_- = \sum_{s\in\mathcal{X}_+} e^{-\beta\mathcal{E}_s}$. $f = 1 + \sum_{i'} \bar{\mu}_{i'} - \sum_{j'} \mu_{j'}$ therefore we can get f in terms of $\bar{\mu}_i$ and μ_j alone

$$f = \frac{1 + \bar{\mu}_i e^{\beta\mathcal{E}_i}\mathcal{Z}_+ - \mu_j e^{\beta\mathcal{E}_j}\mathcal{Z}_-}{x_i e^{\beta\mathcal{E}_i}\mathcal{Z}_+ + x_j e^{\beta\mathcal{E}_j}\mathcal{Z}_- + X_u} \tag{72}$$

We now have f in terms of $\bar{\mu}_i$ and μ_j, and (69) relates these to eachother, so we can solve (64) and (65) simultaneously (using $k = f - \bar{\mu}_i + \mu_j$) to find $\bar{\mu}_i$ and μ_j, and by (67), (68) all Lagrange multipliers. Substituting (67) and (68) into (58) and solving for μ_j gives

$$\mu_j = \frac{k e^{\beta\mathcal{E}_i}(e^{-\beta c} - x_j e^{\beta\mathcal{E}_j+\nu})}{e^{\beta(\mathcal{E}_i-c)} + e^{\beta(\mathcal{E}_j+c)} + (1 - x_i - x_j)e^{\beta(\mathcal{E}_i+\mathcal{E}_j)+\nu}} \tag{73}$$

and similarly for $\bar{\mu}_i$

$$\bar{\mu}_i = \frac{k e^{\beta\mathcal{E}_j}(e^{\beta c} - x_i e^{\beta\mathcal{E}_i+\nu})}{e^{\beta(\mathcal{E}_i-c)} + e^{\beta(\mathcal{E}_j+c)} + (1 - x_i - x_j)e^{\beta(\mathcal{E}_i+\mathcal{E}_j)+\nu}} \tag{74}$$

Using (72) and $k = f - \mu_j + \bar{\mu}_i$ simultaneously solve (73) and (74) to give

$$\bar{\mu}_i = \frac{x_i e^{\nu} - e^{-\beta(\mathcal{E}_i-c)}}{e^{\nu}X_u + \mathcal{Z}_+ e^{\beta c} + \mathcal{Z}_- e^{-\beta c}} \tag{75}$$

$$\mu_j = \frac{e^{-\beta(\mathcal{E}_j+c)} - x_j e^{\nu}}{e^{\nu}X_u + \mathcal{Z}_+ e^{\beta c} + \mathcal{Z}_- e^{-\beta c}} \tag{76}$$

it is simple to check that (67) and 68 result in exactly the same equation for all $\bar{\mu}_{i'}$ and $\mu_{j'}$ but with the corresponding index. Now armed with the explicit form of the Lagrange parameters we have solved the Lagrangian. It is easy to check that for these solutions for $\bar{\mu}_i$, μ_j the Lagrangian simplifies to $\mathcal{L} = \sum_s x_s w(s)$ where $w(s) = 1/\beta \log(f_s e^{\beta\mathcal{E}_s}\mathcal{Z}')$ where the f_i are now of the form

$$f_s = \gamma^{-1}\begin{cases} x_s e^{\nu}, & s \in \mathcal{X}_u \\ e^{\beta(c-\mathcal{E}_s)}, & s \in \mathcal{X}_+ \\ e^{-\beta(c+\mathcal{E}_s)}, & s \in \mathcal{X}_- \end{cases} \tag{77}$$

where

$$\gamma = e^{\nu}X_u + \mathcal{Z}_+ e^{\beta c} + \mathcal{Z}_- e^{-\beta c} \tag{78}$$

Substituting in the above values for f_s into $\mathcal{L} = 1/\beta \sum_s x_s \log(f_s e^{\beta\mathcal{E}_s\mathcal{Z}'})$ gives our final result for work extraction

$$W^{(c)}(\rho) = \frac{1}{\beta}(\log\mathcal{Z}' - \log\gamma) \tag{79}$$

Next we show that γ can be written as $\gamma = \sum_s x_s^0 e^{-\beta\mathcal{E}_s} e^{\beta\theta_s}$ there θ_S is the fluctuation associates with subspace $|s\rangle\langle s|$ which, in the c-bounded distribution, is given by

$$\theta_s = \begin{cases} \frac{1}{\beta}\log(x_s e^{\beta\mathcal{E}_s}) + \frac{\nu}{\beta}, & s \in \mathcal{X}_u \\ +c, & s \in \mathcal{X}_+ \\ -c, & s \in \mathcal{X}_- \end{cases} \tag{80}$$

In general, the c-bounded work is given by the difference between the free energy of the final thermal state and the c-bounded free energy

$$F^{(c)}(\rho) = -\log\sum_s x_s^0 e^{-\beta\mathcal{E}_s} e^{\beta\theta_s} \tag{81}$$

B. Finding the optimal partition

In this Supplementary Note we derive the inequalities for partitioning the state space into positively bounded $\mathcal{X}_+$, negatively bounded $\mathcal{X}_-$ and unbounded $\mathcal{X}_u$ energy levels, as required by our main result. We derive the general set of inequalities for any state and Hamiltonian and give worked through examples of how to find the partition for arbitrary 2 and 3 dimensional systems. Firstly we derive the partition inequalities for work extraction protocols, and

then show that the partition inequalities for work of formation are identical.

The Lagrangian is maximised under the condition that $\bar{\mu}_i$ and μ_j given in (73) and (74) are positive. Therefore if our optimization gives a negative Lagrange parameter it is set to zero, removing the corresponding c-bound. The Lagrange parameters are positive when the following inequalities are satisfied

$$\frac{1}{\beta}\log\left(x_i e^{\beta\mathcal{E}_i}\right) \;>\; c - \frac{\nu}{\beta} \qquad \rightarrow \bar{\mu}_i > 0 \tag{82}$$

$$\frac{1}{\beta}\log\left(x_j e^{\beta\mathcal{E}_j}\right) \;<\; -c - \frac{\nu}{\beta} \qquad \rightarrow \mu_j > 0 \tag{83}$$

where

$$\nu = \frac{\beta}{X_u}\left(\frac{1}{\beta}H_u(\rho) + c(X_- - X_+) - \langle\mathcal{E}_u(\rho)\rangle\right) \tag{84}$$

and we have used $\gamma \geq 0$. Clearly ν depends on how you partition the state space in to positively, negatively and unbounded fluctuations. In the following we derive a set of inequalities that determine the unique partition give ρ and c. The following observations simplify the problem

Lemma 2. For any c-bounded work distribution where bounded fluctuations saturate their bounds, $X_\pm < 1/2$

Proof. Consider a c-bounded work distribution with work values $\{w(s)\}$, average work $W = \sum_s x_s w(s)$, and all work values obey $|w(s) - W| \leq c$. Break the work distribution up into work values that give positive fluctuations $w(i) \geq W$ and negative fluctuations $w(j) < W$. Writing the fluctuations as $\theta_i = w(i) - W$ and $\theta_j = W - w(j)$ and substituting into $W = \sum_s x_s w(s)$ we get

$$\sum_i x_i\theta_i = \sum_j x_j\theta_j \tag{85}$$

which merely states that the average (non-absolute value) fluctuation of a random variable is zero, as is always the case. Take the case that $\sum_{s\in\mathcal{X}_+} x_s > \sum_{s\in\mathcal{X}_-} x_s$ and $X_+ > 1/2$. Clearly $\sum_{s\in\mathcal{X}_-} x_s < 1/2$ so $X_- < 1/2$. If $X_+ \geq 1/2$ then, as bounded fluctuations saturate their bounds, (85) gives the inequality

$$\sum_{s\in\mathcal{X}_-} x_s c < \sum_{s\in\mathcal{X}_+} x_s\theta_s \tag{86}$$

taking the factor of $\sum_{s\in\mathcal{X}_+} x_s$ to the other side we can write the inequality

$$c < \frac{1}{\sum\limits_{s\in\mathcal{X}_-} x_s}\sum_{s\in\mathcal{X}_-} x_s\theta_s \tag{87}$$

The right hand side is a convex sum, and all $\theta_s \geq 0$, therefore at least one $\theta_s > c$ contradicting the fact that the work distribution is c-bounded. Therefore we must have $X_+ < 1/2$. A similar argument for the case $\sum_{s\in\mathcal{X}_+} x_s < \sum_{s\in\mathcal{X}_-} x_s$ gives that $X_- < 1/2$. □

The second observation is that the inequalities (82) and (83) obey a β-ordering hierarchy. The β-odered state, $\rho^{\downarrow\beta}$, is defined as

$$\rho^{\downarrow\beta} = (x_1, x_2, \ldots, x_R) \quad , \quad x_s e^{\beta\mathcal{E}_s} \geq x_{s+1}e^{\beta\mathcal{E}_{s+1}} \tag{88}$$

where $R = \text{rank}(\rho)$ (there is no work value $w_s^{(\infty)}$ associated with $x_s = 0$). The β-ordering gives $w_i^{(\infty)} \geq w_{i+1}^{(\infty)}$. Therefore if $w_i^{(\infty)}$ satisfies (82) then so does $w_{i-1}^{(\infty)}$, and if $w_j^{(\infty)}$ satisfies (83) the so does $w_{j+1}^{(\infty)}$. From this we can deduce that the partition of the state space will look like

$$\rho^{\downarrow\beta} = (\underbrace{x_1, x_2, \ldots, x_k}_{\mathcal{X}_+}, \underbrace{x_{k+1}, \ldots, x_{l-1}}_{\mathcal{X}_u}, \underbrace{x_l, \ldots, x_R}_{\mathcal{X}_-}) \tag{89}$$

Finally, it will be useful to put the inequalities in to the following form. In the following we drop the (∞) superscript from the unbounded work values $w_s^{(\infty)}$. Subscript i will label positively bounded fluctuations and subscript j negatively

bounded fluctuations. (82) and (83) can be written in terms of the unbounded work distribution only (therefore the problem of finding the partition, assuming it exists, is a closed form)

$$W^{(\infty)}(\rho) < X_u(w(i') - c) + \sum_{s\in\mathcal{X}_+} x_s(w(s) - c) + \sum_{s\in\mathcal{X}_-} x_s(w(s) + c) \quad \forall\, i' \in \mathcal{X}_+ \tag{90}$$

$$W^{(\infty)}(\rho) > X_u(w(j') + c) + \sum_{s\in\mathcal{X}_+} x_s(w(s) - c) + \sum_{s\in\mathcal{X}_-} x_s(w(s) + c) \quad \forall\, j' \in \mathcal{X}_- \tag{91}$$

where $W^{(\infty)}(\rho)$ is the unbounded average work $W^{(\infty)}(\rho) = \beta^{-1} \log \mathcal{Z}' + F(\rho)$ and $X_u = 1 - X_+ - X_-$. We now prove that for a given ρ and c there exists a unique partition for which all inequalities (90) and (91) are satisfied.

Lemma 3. Given state ρ, bound value c and inverse temperature β, there is a unique partition of the state space that gives the optimal c-bounded work. For $R = \mathrm{rank}(\rho)$ there are at most $R - 1$ inequalities that must be checked to determine the partition.

Proof. By 2 we know that $X_\pm < 1/2$, therefore $X_u > 0$. The partition obeys the β-ordering hierarchy (89). Starting from x_1 count left to right in $\rho^{\downarrow\beta}$ until you find the furthest x_k s.t. $\sum_{i=1}^{k} x_s < 1/2$. These will form our trial set for the bounded positive fluctuations $\tilde{\mathcal{X}}_+$. Starting from x_R count from right to left until you find the furthest x_l s.t. $\sum_{j=l}^{R} x_j < 1/2$. These form our trial set for the bounded negative fluctuations $\tilde{\mathcal{X}}_-$. If this is indeed the correct partition for the state space, the tightest bounds will be (90) on $w(k)$ and (91) on $w(l)$. The inequalities are

$$(1 - \sum_i x_i - \sum_j x_j)(w(k) - c) + \sum_i x_i(w(i) - c) + \sum_j x_j(w(j) + c) > W(\rho) \tag{92}$$

$$(1 - \sum_i x_i - \sum_j x_j)(w(l) + c) + \sum_i x_i(w(i) - c) + \sum_j x_j(w(j) + c) < W(\rho) \tag{93}$$

Whenever an inequality is satisfied it fixes that fluctuation as being the infinitum of its set. For example, if (92) is satisfied for $w(k)$ then $k \in \tilde{\mathcal{X}}_+$ regardless of $\tilde{\mathcal{X}}_-$. To see this, consider the case that (92) is satisfied for $w(k)$ but (93) isn't for $w(l)$

$$X_u(w(k_-c) + \sum_{i=1}^{k} x_i(w(i) - c) + \sum_{j=l}^{R} x_j(w(j) + c) > W(\rho)$$

$$X_u(w(l) + c) + \sum_{i=1}^{k} x_i(w(i) - c) + \sum_{j=l}^{R} x_j(w(j) + c) > W(\rho)$$

as $w(l)$ doesn't satisfy its bound we have to move further down to $w(l' > l)$. This makes $X_u \to X_u + \sum_{j=l}^{l'} x_j$ and $\sum_{j=l}^{R} x_j(w(j) + c) \to \sum_{j=l}^{R} x_j(w(j) + c) - \sum_{j=l}^{l'} x_j(w(j) + c)$. The left hand side of (92) gains the term

$$+\sum_{j=l}^{l'} x_j(w(k) - c) - \sum_{j=l}^{l'} x_j(w(j) + c) = \sum_{j=l}^{l'} x_j(w(k) - w(j)) \tag{94}$$

As $w(k) \geq w(j)\ \forall\ j = l, \dots, l'$ this term is positive and the new (92) is guaranteed to be satisfied. Therefore we see that there is a unique partition as satisfying an inequalities fixes the corresponding (positive or negative) subspace. There are at most $R - 1$ inequalities that we need to check, i.e. the "worst case" being when the state space is unbounded and we check all $R - 1$ inequalities. □

In summary, the algorithm for determining the partition can be summarised as follows

1. β-order the state $\rho^{\downarrow\beta} = (x_1, \ldots, x_R)$ where $x_s^{\beta\mathcal{E}_s} \geq x_{s+1}^{\beta\mathcal{E}_{s+1}}$ and $R = \mathrm{rank}(\rho)$. This ordering gives the unbounded work distribution in descending order $(w(1), \ldots, x_R)$ where $w(s) = \beta^{-1}\log(x_s e^{\beta\mathcal{E}_s}\mathcal{Z}')$ and $w(s) \geq w(s+1)$

2. Take the trial partition where you maximise $X_+ = \sum_{i=1}^k x_i$ under the condition $X_+ < 1/2$ and $X_- = \sum_{j=l}^R x_j$, $X_- < 1/2$. Check inequalities

$$X_u(w(k) - c) + \sum_{i=1}^{k} x_i(w(i) - c) + \sum_{j=l}^{R} x_j(w(j) + c) > W(\rho) \tag{95}$$

$$X_u(w(l) + c) + \sum_{i=1}^{k} x_i(w(i) - c) + \sum_{j=l}^{R} x_j(w(j) + c) < W(\rho) \tag{96}$$

3. If (95) is satisfied x_k fixes $X_+ = \sum_{i=1}^k x_i$ and similar for (96). Otherwise we perform the next set of inequalities lower in the hierarchy, with $X_+ = \sum_{i=1}^{k-1} x_i$ if (95) is not satisfied and/or $\sum_{j=l+1}^R x_j$ if (96) is not satisfied. We repeat this process until we find a pair of inequalities that are simultaneously satisfied, fixing $\tilde{\mathcal{X}}_\pm = \mathcal{X}_\pm$, requiring at most $R-1$ inequalities to be checked.

Case: d=2. This is the simplest case, as either $x_1 > 1/2$ or $x_2 > 1/2$ or they both $= 1/2$. In the first case we bound the negative fluctuation, and there is a single bound to check

$$w(2) < W^{(\infty)}(\rho) - c \tag{97}$$

and in the second case we bound the positive fluctuation if

$$w(1) > W^{(\infty)}(\rho) + c \tag{98}$$

and if $x_1 = x_2 = 1/2$ when the two fluctuations must be equal (as the average of the positive fluctuations = the average of the negative fluctuations) and we can choose to bound one or the other, giving the same free energy.

In the following section we show that the same algorithm is used for determining the partition for the c-bounded work of formation. Note that, unless all fluctuations are unbounded $W^{(c)}(\rho) < W^{(\infty)}(\rho)$ and, as shown in the next section, $W_{\mathrm{F}}^{(c)}(\rho) > W_{\mathrm{F}}^{(\infty)}(\rho)$.

IV. SUPPLEMENTARY NOTE 4

In this Supplementary Note we derive the c-bounded minimal work of formation $W_{\mathrm{F}}^{(c)}$.

A. determining the optimal work of formation map

Assume there exists some optimal choice of $\{t(s'|s), w(s'|s)\}$ that minimize the work cost whilst obeying the c-bounds and microscopic reversibility (29). We start in the thermal state with $x_s = 1/\mathcal{Z}e^{-\beta\mathcal{E}_s}$ and end the protocol in state ρ with probabilities $x_{s'}$. The average work is

$$W = \sum_{ss'} \frac{1}{\mathcal{Z}} e^{-\beta\mathcal{E}_s} t(s'|s) w(s'|s) \tag{99}$$

where the t_{ij} must obey (26), (27) and (28).

Lemma 4. We can always choose new protocol parameters $\tilde{t}(s') = x_{s'}$ and $\tilde{w}_{s'} = \sum_s \frac{t(s'|s)e^{-\beta\mathcal{E}_s}}{x_{s'}\mathcal{Z}} w(s'|s)$ that give the same average work as the optimal protocol and obey all the necessary constraints

Proof. Clearly these choices of $\tilde{t}(s')$ satisfy $\sum_{s'} \tilde{t}(s') = 1$ and $\sum_s 1/\mathcal{Z}e^{-\beta\mathcal{E}_s}\tilde{t}(s') = x_{s'}$, and the average work is given by

$$\tilde{W} = \sum_{ss} 1/\mathcal{Z}e^{-\beta\mathcal{E}_s}\tilde{t}(s')\tilde{w}_{s'} = \sum_{s'} \tilde{t}(s')\tilde{w}_{s'} \tag{100}$$
$$= \sum_{ss'} x_{s'} \frac{t(s'|s)e^{-\beta\mathcal{E}_s}}{\mathcal{Z}x_{s'}} w(s'|s) = W$$

Note that $\sum_s \frac{t(s'|s)e^{-\beta\mathcal{E}_s}}{x_{s'}\mathcal{Z}} = 1$ so $\tilde{w}_{s'}$ is a convex sum of the $w(s'|s)$. The reversibility inequality (29) demands that

$$\sum_s \tilde{t}(s')e^{\beta(\tilde{w}_{s'}-\Delta\mathcal{E}_{ss'})} \leq 1 \tag{101}$$

which we can see to be true as the LHS is

$$\begin{aligned}
\text{LHS } &= x_{s'}e^{\beta(\tilde{w}_{s'}+\mathcal{E}_{s'})}\sum_s e^{-\beta\mathcal{E}_s} \\
&= x_{s'}\mathcal{Z}e^{\beta(\tilde{w}_{s'}+\mathcal{E}_{s'})} \\
&= x_{s'}\mathcal{Z}e^{\beta\mathcal{E}_{s'}}e^{\beta\sum_i \frac{t(s'|s)e^{-\beta\mathcal{E}_s}}{x_{s'}\mathcal{Z}}w(s'|s)} \\
&\leq x_{s'}\mathcal{Z}e^{\beta\mathcal{E}_{s'}}\sum_s \frac{t(s'|s)e^{-\beta\mathcal{E}_s}}{x_{s'}\mathcal{Z}}e^{\beta w(s'|s)} \\
&= \sum_s t(s'|s)e^{\beta(w(s'|s)-\Delta\mathcal{E}_{ss'})} \leq 1
\end{aligned}$$

where in the fourth step we have used the convexity of the exponential function and in the last step we have used the fact that the optimal protocol defined by $\{t(s'|s), w(s'|s)\}$ is reversible.
Clearly the new protocol obeys the c-bounds as $\tilde{w}_{s'}$ is a convex combination of $w(s'|s)$ and the average of the work distribution is the same for both maps, therefore the spread of $\tilde{w}_{s'}$ about the average is less than or equal to that of $w(s'|s)$. □

Substituting $t(s'|s) \to \tilde{t}(s') = x_{s'}$ and $w(s'|s) \to \tilde{w}_{s'}$ into the Lagrangian and simplifying gives

$$\begin{aligned}
\mathcal{L} = &\sum_{s'} f_{s'}w(s') + \sum_{s'}\lambda_{s'}\left(1 - x_{s'}e^{\beta\mathcal{E}_{s'}}\mathcal{Z}e^{\beta w(s')}\right) \\
&+ c\sum_{s'}(\mu_{s'} + \bar{\mu}_{s'})
\end{aligned}$$

where we have dropped the tilde from $\tilde{w}_{s'}$ and $f_{s'} = x_{s'}(1+f) - (\bar{\mu}_{s'} - \mu_{s'})$ and $f = \sum_{s'}(\bar{\mu}_{s'} - \mu_{s'})$. $\bar{\mu}$ give the bounds on positive fluctuations and μ give the bounds for negative fluctuations. Maximizing w.r.t $w(s')$ and $\lambda_{s'}$ gives

$$\lambda_{s'} \ = \ \max\{\frac{f_{s'}}{\beta}\ ,\ 0\,\} \tag{102}$$

$$w(s') \ = \ -\frac{1}{\beta}\log\left(\frac{\beta\lambda_{s'}x_{s'}e^{\beta\mathcal{E}_{s'}}\mathcal{Z}}{f_{s'}}\right) \tag{103}$$

$$\ = \ -\frac{1}{\beta}\log\left(x_{s'}e^{\beta\mathcal{E}_{s'}}\mathcal{Z}\right)\ , \quad f_{s'} \neq 0 \tag{104}$$

which is the work value given in the unbounded cases (it contains no $\bar{\mu}_j, \mu_j$ terms). Therefore all fluctuations with $f_{s'} > 0$ are unbounded, with $\mu_{s'}(\bar{\mu}_{s'}) = 0$, giving $f_{s'} = x_{s'}$ if unbounded or 0 if bounded. The Lagrangian reduces to

$$\mathcal{L} = -\frac{1}{\beta}\sum_{s'} f_{s'}\log\left(x_{s'}e^{\beta\mathcal{E}_{s'}}\mathcal{Z}\right) + c\sum_{s'}\left(\bar{\mu}_{s'} + \mu_{s'}\right) \tag{105}$$

Which we can now re-write in terms of bounded and unbounded fluctuations. Index all fluctuations with $f_{s'} > 0$ with $s' \in \mathcal{X}_u$, and all those with $f_{s'} = 0$ with $s' \in \mathcal{X}_+$. The Lagrangian becomes

$$\mathcal{L} = -\frac{1}{\beta}\sum_{s'\in\mathcal{X}_u} x_{s'}(1+f)\log\left(x_{s'}e^{\beta\mathcal{E}_{s'}}\mathcal{Z}\right) + c\sum_{s'\in\mathcal{X}_+}\left(\bar{\mu}_s + \mu_s\right) \tag{106}$$

$f_s = 0\ \forall s' \in \mathcal{X}_+$ gives

$$\bar{\mu}_{s'} - \mu_{s'} = x_{s'}(1+f) \tag{107}$$

Summing over $s \in \mathcal{X}_+$ and solving for $f = \sum_{k'}(\bar{\mu}_{k'} - \mu_{k'})$ gives

$$f = \frac{X_c}{1-X_c} \tag{108}$$

where $X_c = \sum_{s' \in \mathcal{X}_+} x_s$. For a given bounded fluctuation only one of the μ Lagrange parameters is non-zero, corresponding to if the fluctuation is positive or negative. Using the above result gives

$$\bar{\mu}_k = x_k(1 + \frac{X_c}{1 - X_c}) = \frac{x_k}{1 - X_c} \tag{109}$$

$$\mu_l = -\frac{x_l}{1 - X_c} \tag{110}$$

The Lagrange parameters are restricted to being positive, $\mu_s \geq 0$ and $\bar{\mu}_s \geq 0 \ \forall \ s$. Therefore as $\mu_s = -x_s/(1 - X_c)$ which is ≤ 0, therefore all $\mu_s = 0$ and we never bound negative fluctuations.

Using $X_u = 1 - X_c$ we arrive at the c-bounded optimal work of formation

$$W_F^{(c)} = \frac{1}{X_u}\left(-\frac{1}{\beta}\sum_{s' \in \mathcal{X}_u} x_{s'} \log\left(x_{s'} e^{\beta \mathcal{E}_{s'}} \mathcal{Z}\right) + c\, X_c\right) \tag{111}$$

This is exactly the work cost when we calculate the c-bounded work of formation in the following way. Take the optimal unbounded work distribution for forming a state $(w(1), \ldots, w(d))$ with average $W^{(\infty)}(\rho)$. Equation (111) is given by the solution to the equation

$$W = \sum_{s \in \mathcal{X}_u} x_s w(s) + \sum_{s \in \mathcal{X}_+} x_s (W + c)$$

$$\therefore W \underbrace{(1 - \sum_{s \in \mathcal{X}_+} x_s)}_{X_u} = \sum_{s \in \mathcal{X}_u} x_s w(s) + cX_+$$

$$\therefore W = \frac{1}{X_u}\left(\sum_{s \in \mathcal{X}_u} x_s w(s) + cX_+\right)$$

I.e we simply take the optimal unbounded work distribution and if the largest positive fluctuation breaks $w(i) - W \leq c$ we replace it with $W' + c$, recalculating the average each time.

B. finding the optimal partition for state formation

We now show this algorithm for finding the partition is identical to the algorithm derived in Supplementary Note 2 for work extraction, in the case of positive fluctuation bounds only.

Theorem 3. The partition of the state space that gives the c-bounded work of formation is found using the algorithm described in Lemma 3 but bounding only positive fluctuations.

Proof. Take the final state ρ' and β-order it

$$\rho'^{\downarrow\beta} = (x_1, \ldots x_R) \quad , \quad x_{s'} e^{\beta \mathcal{E}_{s'}} \geq x_{s'+1} e^{\beta \mathcal{E}_{s'+1}} \tag{112}$$

where $R = \text{rank}(\rho)$ (we can discount work values in the unbounded work distribution associated with $x_s = 0$ as they have no probability of being observed). The unbounded work distribution obeys the inverse β-ordering as $w(s') = -\beta^{-1} \log(x_{s'} e^{\beta \mathcal{E}_{s'}} \mathcal{Z})$, therefore $w(1) \leq w(2) \leq \cdots \leq w(R)$. For a correct partition where $X_+ = \sum_{s'=k}^{R} x_i$ we require that $w(s') \leq W_F(\rho')^{(c)} + c \ \forall \ s < k$. As with the c-bounded work extraction protocol the β-ordering puts these inequalities into a hierarchy, with the bound for $w(k-1)$ being the tightest. As all bounds must be satisfied we need only check the tightest one. For the aforementioned partition the tightest bound is

$$w(k-1) \leq \frac{1}{\sum\limits_{i=k}^{R} x_i}\left(W^{(\infty)} - \sum_{s'=k}^{R} x_{s'} w(s') + c \sum_{s'=k}^{R} x_{s'}\right) \tag{113}$$

which can be re-arranged to give

$$(1 - \sum_{s'=k}^{R} x_{s'}) w(k-1) + \sum_{s'=k}^{R} x_{s'} w(s') \leq W^{(\infty)} + c \tag{114}$$

which is simply the inequality used for checking a partition of the state space in the c-bounded work extraction protocol, in the case that there are no negative fluctuations that saturate their bounds (95). It can be simplified further to

$$\sum_{s'=1}^{k-1} x_{s'}(w(k-1) - w(s')) \leq c \tag{115}$$

So the partition is defined by the largest k s.t.

$$\sum_{s'=1}^{k} x_{s'}(w(k) - w(s')) > c \tag{116}$$

$$\sum_{s'=1}^{k-1} x_{s'}(w(k-1) - w(s')) \leq c \tag{117}$$

All $w(s)$ with $s \geq k$ are bounded and all with $s < k$ are unbounded. Once again we have at most $R-1$ inequalities to check. I.e. starting from $k = 2$ we check (116) for $k = 2, 3, \ldots$. □

V. SUPPLEMENTARY NOTE 5

A. recovering standard and single-shot regimes

Theorem 4. $\lim_{c\to 0} W^{(c)}(\rho) = W_{\min}(\rho)$

Proof. As $c \to 0$ the inequalities that determine if a fluctuation saturates its bound (82) and (83) become

$$\frac{1}{\beta}\log\left(x_s e^{\beta\mathcal{E}_s}\right) \geq -\nu/\beta \to s \in \mathcal{X}_+$$
$$\frac{1}{\beta}\log\left(x_s e^{\beta\mathcal{E}_s}\right) \leq -\nu/\beta \to s \in \mathcal{X}_-$$

All fluctuations satisfy one of these bounds, except in the case that $x_s = 0$ (i.e. there is no fluctuation associated with state $|s\rangle$), therefore $X_u \to 0$. $\gamma = X_u e^{\nu} + \mathcal{Z}_+ e^{\beta c} + \mathcal{Z}_- e^{-\beta c} \to \mathcal{Z}_+ + \mathcal{Z}_-$ which can be formulated as

$$\gamma = \sum_s x_s^0 e^{-\beta\mathcal{E}_s} \tag{118}$$

therefore $W^{(c)}(\rho)$ becomes

$$\begin{aligned}\lim_{c\to 0} W^{(c)}(\rho) &= \frac{1}{\beta}\left(\log\mathcal{Z}' - \log\sum_s x_s^0 e^{-\beta\mathcal{E}_s}\right) \\ &= W_{\min}(\rho)\end{aligned} \tag{119}$$

□

Theorem 5. $\lim_{c\to 0} W_F^{(c)}(\rho) = W_F^{\min}(\rho)$

Proof. Following the partition algorithm derived in Lemma 3, clearly when $c \to 0$ we bound all but the most negative fluctuation(s), those with value $w(1)$, as this gives the only positively bounded work distribution for which all work values are $\leq W_F^{(0)}$. Any other choice of partition would require both negative and positive bound saturation, which contradicts (109) being positive. Therefore $W_F^{(0)}$ is given by

$$W_F^{(0)} = \min_s\left\{-\frac{1}{\beta}\log\left(x_s e^{\beta\mathcal{E}'_s}\mathcal{Z}\right)\right\} = -\frac{1}{\beta}\log\left(x_1 e^{\beta\mathcal{E}'_1}\mathcal{Z}\right) \tag{120}$$

where $\rho'^{\downarrow\beta} = (x_1, \ldots, x_R)$. We can interpret

$$w(1) = -\frac{1}{\beta}\log\left(x_1 e^{\beta\mathcal{E}'_1}\mathcal{Z}\right) \tag{121}$$

$-w(1)$ is the "on ramp" (the first segment) of the Lorenz curve of state ρ' (see Figure 1 and [2]). It therefore gives the work that can be extracted from the "thermally sharp state", where all segments either have the same gradient or zero gradient, that just thermomajorizes ρ', see figure 1 below and also [2]. This can also be interpreted as the upper bound to the work that can be in-deterministically extracted from ρ', and this is precisely the single-shot work of formation. □

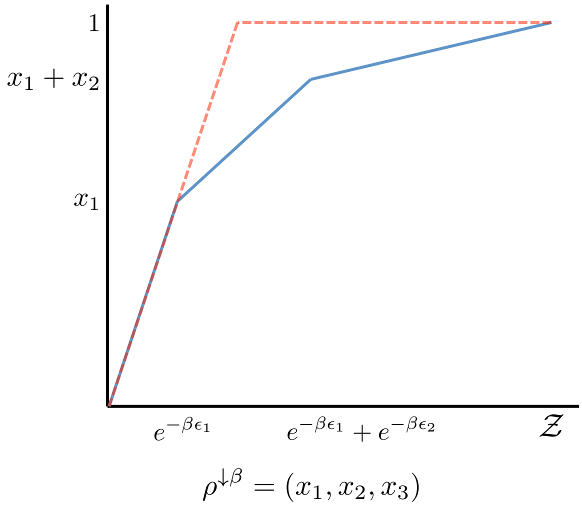

FIG. 1: Lorenz curve depicting work of formation of a state. Figure shows the thermally sharp state whose single-shot work content gives the work of formation of state ρ'. This also represents the maximal average work that can be extracted from ρ'

B. bounds on c-bounded work

Lemma 5. $W^{(c)}(\rho) \leq W_{\min}(\rho) + c$. $c \in [0, c_{\text{crit}}]$, therefore any gain in work extracted over the single-shot work context of ρ requires fluctuations that are at least as large as the increase in extracted work

Proof. For $c = 0$ we get that $W_{c=0}(\rho) = W_{\min}(\rho)$. $W^{(c)}$ increases as we increase c from 0. We show $W^{(c)}(\rho)$ grows sub-linearly with c, i.e. that

$$\frac{\partial W^{(c)}(\rho)}{\partial c} \leq 1 \quad \forall\, c \tag{122}$$

which implies that $|W^{(c)}(\rho) - W_{\min}(\rho)| \leq c$. Using (79) the derivative of $W^{(c)}(\rho)$ with respect to c is

$$\frac{\partial W^{(c)}(\rho)}{\partial c} = -\frac{1}{\beta\gamma}\frac{\partial \gamma}{\partial c} = \frac{1}{\gamma}\left((X_+ - X_-)e^{\nu} - \mathcal{Z}_+ e^{\beta c} + \mathcal{Z}_- e^{-\beta c}\right) \tag{123}$$

where $\gamma = X_u e^{\nu} + \mathcal{Z}_+ e^{\beta c} + \mathcal{Z}_- e^{-\beta c}$. For this to be greater than 1 we require that

$$(X_+ - X_-)e^{\nu} - \mathcal{Z}_+ e^{\beta c} + \mathcal{Z}_- e^{-\beta c} > X_u e^{\nu} + \mathcal{Z}_+ e^{\beta c} + \mathcal{Z}_- e^{-\beta c} \tag{124}$$

As $\mathcal{Z}_\pm e^{\pm\beta c} \geq 0$ and $e^{\nu} \geq 0$ this cannot be satisfied unless

$$X_+ - X_- > X_u \overset{\therefore}{\rightarrow} X_+ > \frac{1}{2} \tag{125}$$

where we have used $X_u = 1 - X_+ - X_-$. We now show that this is never true for c-bounded work distributions. Using $W^{(c)}(\rho) = \sum_s x_s \theta_s$, where $\theta_s = w(s) - W^{(c)}(\rho)$ is the fluctuation associated with work value $w(s)$, gives $\sum_s x_s \theta_s = 0$. Divide the fluctuations for the c-bounded protocol θ_s into positive $\theta_\alpha \geq 0$ where $\alpha \in \mathcal{X}_+$ and negative $\theta_\beta < 0$ where $\beta \in \mathcal{X}_-$, giving

$$\sum_\alpha x_\alpha |\theta_\alpha| = \sum_\beta x_\beta |\theta_\beta| \tag{126}$$

according to (56) and (57), bounded fluctuations saturate their bounds. Take the subset of the positive fluctuations that saturate their bounds as $\theta_{\alpha'}$. (126) becomes

$$cX_+ + \sum_{\alpha \neq \alpha'} x_\alpha |\theta_\alpha| = \sum_\beta x_\beta |\theta_\beta| \tag{127}$$

Dividing both sides by $\sum_\beta x_\beta$ gives

$$\frac{\sum\limits_\beta x_\beta |\theta_\beta|}{\sum\limits_\beta x_\beta} = \frac{cX_+ + \sum\limits_{\alpha \neq \alpha'} x_\alpha |\theta_\alpha|}{\sum\limits_\beta x_\beta} \tag{128}$$

the right hand side is strictly larger than c as $X_+ > 1/2$ and therefore $\sum_\beta x_\beta < 1/2$, and $\sum_{\alpha\neq\alpha'} x_\alpha|\theta_\alpha| \geq 0$. The left hand side is a convex sum with all $|\theta_\beta| \geq 0$, therefore at least one $|\theta_\beta|$ must be larger than c. Therefore in a c-bounded protocol $X_+ \leq 1/2$. □

Lemma 6. $\left|W_F^{(c)}(\rho')\right| \geq |W_{\max}(\rho')| - c$. $c \in [0, c_{\text{crit}}]$, therefore any gain in work extracted over the single-shot work of formation of ρ' requires fluctuations that are at least as large as the increase in extracted work

Proof. if $\left|W_F^{(c)}(\rho')\right| < |W_{\max}(\rho')| - c$ then, taking $w(1) = \max\{w(s')\} = \max\{\beta^{-1}\log x_{s'} e^{\beta\epsilon_{s'}}\mathcal{Z}\} = |W_{\max}(\rho')|$ when we require that

$$\frac{1}{X_u}\left(\sum_{s'\in\mathcal{X}_u} w(s') + c(1-X_u)\right) + c < w(1) \tag{129}$$

which requires that

$$c < \sum_{s'\in\mathcal{X}_u} (w(1) - w(s')) \tag{130}$$

which contradicts (115) for any partition □

C. c-bounded work obeys the Jarzinski equality

In this Supplementary Note we show that all c-bounded work distributions obey the Jarzinski equality. The equality is given in the case that a system begins in the thermal state of an initial Hamiltonian H_i and the Hamiltonian is transformed to H_f, causing the state to evolve to a state that is, potentially, out of equilibrium with the final Hamiltonian. The equality is stated as

$$\langle e^{-\beta w}\rangle = e^{-\beta\Delta F} \tag{131}$$

where w is the work random variable (i.e. the work values associated with the protocol), with the convention that they are positive if the work is done on the system, and ΔF is the free energy difference between the equilibrium states of $H_{\mathcal{S}}$ and $H'_{\mathcal{S}}$, given by

$$\Delta F = \frac{1}{\beta}\log\left(\frac{\mathcal{Z}}{\mathcal{Z}'}\right) \tag{132}$$

Theorem 6. Any protocol that saturates all reversibility constraints immediately satisfies the Jarzinski equality

Proof. If a protocol, characterised by $\{t(s'|s), w(s'|s)\}$, saturates all reversibility constraints then

$$\sum_s t(s'|s)e^{\beta(w(s'|s)-\mathcal{E}_s+\mathcal{E}_{s'})} = 1 \tag{133}$$

Dividing by $\mathcal{Z}$ and re-arranging gives

$$\sum_s \frac{e^{-\beta\mathcal{E}_s}}{\mathcal{Z}} t(s'|s)e^{\beta w(s'|s)} = \frac{e^{-\beta\mathcal{E}_{s'}}}{\mathcal{Z}} \tag{134}$$

if we sum over s', the LHS of this is equivalent to $\langle e^{\beta w(s'|s)}\rangle$ with the initial state being thermal w.r.t $H_{\mathcal{S}}$, $x_s = \mathcal{Z}^{-1}e^{-\beta\mathcal{E}_s}$, and the RHS becomes $\mathcal{Z}'/\mathcal{Z}$, which is equal to $e^{\beta\Delta F}$. Note that we use the convention of positive work for work extracted from the system, hense our $w(s'|s) = -w(s)$ are the negatives of the work values given in the Jarzinski equality.

□

Theorem 7. The work distributions associated with $W^{(c)}(\rho)$ and $W_F^{(c)}(\rho)$ obey the Jarzinski equality.

First we cover the case of work extraction, $W^{(c)}(\rho)$. The work values are give by

$$w(s) = \frac{1}{\beta}\log(f_s e^{\beta\mathcal{E}_s}\mathcal{Z}') \tag{135}$$

and the $t(s'|s)$ are given by

$$t(s') = \frac{e^{-\beta \mathcal{E}'_{s'}}}{\mathcal{Z}'} \tag{136}$$

as derived in Supplementary Note 2. Note that

$$\sum_s f_s = 1 \tag{137}$$

The LHS of the reversibility constraints are

$$\begin{aligned} &= \sum_s \frac{e^{-\beta \mathcal{E}_{s'}}}{\mathcal{Z}'} e^{\beta(w(s) - \mathcal{E}_s + \mathcal{E}_{s'})} \\ &= \sum_s \frac{1}{\mathcal{Z}'} e^{\log(f_s \mathcal{Z}'))} \\ &= \sum_s f_s = 1 \end{aligned}$$

and can similarly be shown for $W^{(c)}(\rho)$.

VI. SUPPLEMENTARY NOTE 6

In this Supplementary Note we calculate the c-bounded Carnot efficiency for a single qubit quantum engine. The engine operates by moving a qubit $\rho = x\,|0\rangle\langle 0| + (1-x)\,|1\rangle\langle 1|$ with Hamiltonian $H_s = \mathcal{E}\,|0\rangle\langle 0|$ between two baths with inverse temperature β_H and β_C, with $\beta_H < \beta_C$. The engine cycle begins with the qubit in thermal equilibrium with the cold bath. It is then placed in contact with the hot bath, and extracting work by allowing the state to equilibrate. The final step in the cycle is to return the qubit to the cold bath, allowing it to equilibrate and extracting work.
Case: $\mathbf{c} \to \infty$. Following the proof given in [8], we show that in the case that fluctuations are unbounded it is possible to reach Carnot efficiency. The equilibrium state of the qubit if given by

$$\rho_{H,C} = \frac{1}{Z_{H,C}} e^{-\beta_{H,C}\mathcal{E}}\,|0\rangle\langle 0| + \frac{1}{Z_{H,C}}\,|1\rangle\langle 1| \tag{138}$$

where $\mathcal{Z}_{H,C} = e^{-\beta_{H,C}\mathcal{E}} + 1$. When ρ_C is placed in thermal contact with the hot bath we can extract a maximal average work given by the free energy difference

$$\begin{aligned} F(\rho_C, \beta_H) - F(\rho_H, \beta_H) = &\frac{1}{\beta_H} \log\left(\frac{\mathcal{Z}_H}{\mathcal{Z}_C}\right) \\ &+ \mathrm{tr}\left(H_s \rho_c\right)\left(\frac{\beta_H - \beta_C}{\beta_H}\right) \end{aligned} \tag{139}$$

where $\mathrm{tr}\left(H_s \rho_C\right) = \mathcal{Z}_C^{-1} e^{-\beta_C \mathcal{E}} \mathcal{E}$ and work values

$$\begin{aligned} w_{0,H} &= \frac{1}{\beta_H} \log\left(\frac{\mathcal{Z}_H}{\mathcal{Z}_C} e^{\mathcal{E}(\beta_H - \beta_C)}\right) \\ w_{1,H} &= \frac{1}{\beta_H} \log\left(\frac{\mathcal{Z}_H}{\mathcal{Z}_C}\right) \end{aligned} \tag{140}$$

where we have used the result for the optimal unbounded work extraction protocol $w(s) = \beta^{-1} \log x_s e^{\beta \mathcal{E}_s} \mathcal{Z}$. Returning the qubit to the cold bath we extract work

$$\begin{aligned} F(\rho_H, \beta_C) - F(\rho_C, \beta_C) = &\frac{1}{\beta_C} \log\left(\frac{\mathcal{Z}_C}{\mathcal{Z}_H}\right) \\ &+ \mathrm{tr}\left(H_s \rho_H\right)\left(\frac{\beta_C - \beta_H}{\beta_C}\right) \end{aligned} \tag{141}$$

with work values

$$\begin{aligned} w_{0,C} &= \frac{1}{\beta_C} \log\left(\frac{\mathcal{Z}_C}{\mathcal{Z}_H} e^{\mathcal{E}(\beta_C - \beta_H)}\right) \\ w_{1,C} &= \frac{1}{\beta_C} \log\left(\frac{\mathcal{Z}_C}{\mathcal{Z}_H}\right) \end{aligned} \tag{142}$$

where $\mathrm{tr}\,(H_s\rho_H) = \mathcal{Z}_H^{-1} e^{-\beta_H \mathcal{E}} \mathcal{E}$. The total work extracted in one cycle is

$$W_{\mathrm{tot}} = \left(\frac{1}{\beta_H} - \frac{1}{\beta_C} \right) (S_H - S_C) \tag{143}$$

where $S_{H,C} = -\log \mathcal{Z}_{H,C} - \beta_{H,C} \mathrm{tr}\,(H_s \rho_{H,C})$ is the Von Neumann entropy of the Gibbs state with inverse temperature $\beta_{H,C}$. Applying the first law of thermodynamics $\Delta U = Q - W$ to the first step (extracting work from the hot bath), we get

$$\mathrm{tr}\,(H_s\rho_H) - \mathrm{tr}\,(H_s\rho_C) = Q_H - (F(\rho_C, \beta_H) - F(\rho_H, \beta_H)) \tag{144}$$

which simplifies to

$$Q_H = \frac{1}{\beta_H} (S_H - S_C) \tag{145}$$

where Q_H is the heat flow out of the hot bath. The efficiency is given by the ratio of the total work to Q_H, giving

$$\frac{W_{\mathrm{tot}}}{Q_H} = 1 - \frac{\beta_H}{\beta_C} \tag{146}$$

which is the Carnot efficiency.

Case: c finite.

$\mathcal{E} > 0$ implies that $x < 1/2$ for all thermal states of ρ, therefore by the partitioning algorithm derived in Supplementary Note 4, the least likely fluctuation $w_0^{(c)}$ associated with subspace $|0\rangle$ is the only fluctuation that can be bounded. Using the result of Supplementary note 3, the c-bounded work content for a qubit $\rho^{\downarrow\beta} = (x_1, x_2)$ is given by

$$W^{(c)}(\rho) = \frac{1}{\beta} \log \mathcal{Z} + \begin{cases} F(\rho)\,, & x_1 e^{\beta \mathcal{E}_1} \leq e^{\beta(F(\rho)+c)} \\ & x_2 e^{\beta \mathcal{E}_2} \geq e^{\beta(F(\rho)-c)} \\ F_1^{(c)}(\rho)\,, & w(0) < W(\rho) - c \\ F_2^{(c)}(\rho)\,, & w(0) > W(\rho) + c \end{cases} \tag{147}$$

where $F_1^{(c)}(\rho) = -\beta^{-1} \log \left(e^{-c\beta + \frac{c\beta}{1-x}} + e^{-c\beta - \mathcal{E}\beta} \right)$ and $F_2^{(c)}(\rho) = -\beta^{-1} \log \left(e^{c\beta - \frac{c\beta}{1-x}} + e^{c\beta - \mathcal{E}\beta} \right)$, $W(\rho)$ is the unbounded work given by the free energy, and $w(0) = \beta^{-1} \log(x e^{\beta \mathcal{E}} \mathcal{Z})$. In the first step, putting the qubit in contact with the hot bath, the work we extract is bounded negatively if $w_{0,H} < F(\rho_C, \beta_H) - F(\rho_H, \beta_H) - c$, which gives the inequality

$$\frac{\mathcal{E}}{\mathcal{Z}_C} \left(\frac{\beta_C - \beta_H}{\beta_H} \right) > c \tag{148}$$

note that $\beta_C > \beta_H$ therefore the LHS of (148) is positive. In order to break the positive fluctuation bound we would require -LHS $> c$ which is impossible for positive c, therefore in the first part of the engine cycle the work extracted is either unbounded or negatively bounded. For the second cycle, re-equilibriating the qubit with the cold bath, the inequality that implies a positively bounded work is given by $w_{0,C} > F(\rho_H, \beta_C) - F(\rho_C, \beta_C) + c$, which simplifies to

$$\frac{\mathcal{E}}{\mathcal{Z}_H} \left(\frac{\beta_C - \beta_H}{\beta_C} \right) > c \tag{149}$$

Again the LHS is positive, and we would require -LHS $> c$ in the case of a negatively bounded protocol. Therefore on the second part of the cycle the work is either unbounded or positively bounded. Also note that as $\beta_H < \beta_C$ and therefore $\mathcal{Z}_C < \mathcal{Z}_H$, satisfying inequality (148) implies inequality (149) is also satisfied. There are therefore three cases, 1) the work is unbounded and we can achieve Carnot efficiency, 2) the work extracted from the hot bath is negatively bounded, and 3) 2 and the work extracted from the cold bath is positively bounded.

the c-bounded work extracted from the hot bath, in the case that inequality (148) is satisfied, is given by

$$W_1^{(c)} = \frac{1}{\beta_H} \log \left(\frac{\mathcal{Z}_H}{e^{c\beta_H} \mathcal{Z}_C + e^{-\beta_H \mathcal{E}}} \right) + c \tag{150}$$

where we have used the expressions in (147). If inequality (149) is satisfied for the second part of the cycle, then we get

$$W_2^{(c)} = \frac{1}{\beta_C} \log \left(\frac{\mathcal{Z}_C}{e^{-c\beta_C} \mathcal{Z}_H + e^{-\beta_C \mathcal{E}}} \right) - c \tag{151}$$

Using $Q_H = \Delta U + W_1^{(c)}$, the efficiency is given by

$$\eta_1^{(c)} = \frac{W_1^{(c)} + F(\rho_H, \beta_C) - F(\rho_C, \beta_C)}{\Delta U + W_1^{(c)}} \tag{152}$$

if inequality (148) is satisfied and

$$\eta_2^{(c)} = \frac{W_1^{(c)} + W_2^{(c)}}{\Delta U + W_1^{(c)}} \tag{153}$$

if inequalities (148) and (149) are satisfied. As the temperature difference between hot and cold baths increases, the efficiency increases. In the unbounded case, the Carnot efficiency is bounded from above by 1. As $\beta_H/\beta_C \to 0$, inequality (149) cannot be satisfied for any finite c. To find the upper bound of $\eta_1^{(c)}$ we make the change of variables $\beta_C/n = \beta_H$, i.e. $T_H = nT_C$ and n is the ratio of the two bath temperatures. We then take $\beta_H \to \infty$ and $n \to \infty$.

$$\lim_{n\to 0}\left(\lim_{\beta_H\to\infty}\left(\eta_1^{(c)}\right)\right) = 1 - \frac{\mathcal{E}}{2(2c+\mathcal{E})} \tag{154}$$

Of course as $\mathcal{E} \to 0$ the efficiency tends to 1, but this is because we are no longer extracting any work (as the two Gibbs states are identical). As $c \to \infty$ we recover the Carnot efficiency upper bound, although for any finite c we can never reach an efficiency of 1. Therefore all realistic engines of this form have an upper bound to their efficiency defined by their energy gap and fragility c. This represents a general physical upper bound on the efficiency of a the qubit engine, given by its ability to withstand fluctuations. Also notice that as $c \to 0$ the maximal efficiency is bounded from below by 1/2. For $c = 0$ the efficiency is zero, as no work can be extracted. Therefore we observe, in this model at least, a genuine discontinuity between the capabilities of a thermal machine in the single-shot regime. Allowing for arbitrarily small fluctuations in principle will allow the engine to reach a maximum efficiency that is bounded from below by 1/2. But if we demand that the work is deterministic, the efficiency is zero.

Supplementary References

---

[1] Lluis Masanes and Jonathan Oppenheim. A derivation (and quantification) of the third law of thermodynamics. Preprint at https://arxiv.org/abs/1412.3828, 2014.

[2] Michał Horodecki and Jonathan Oppenheim. Fundamental limitations for quantum and nanoscale thermodynamics. *Nature communications*, 4, 2013.

[3] Johan Aberg. Fully quantum fluctuation theorems. Preprint at https://arxiv.org/abs/1601.01302, 2016.

[4] Matteo Lostaglio, David Jennings, and Terry Rudolph. Description of quantum coherence in thermodynamic processes requires constraints beyond free energy. *Nature communications*, 6, 2015.

[5] Matteo Lostaglio, Kamil Korzekwa, David Jennings, and Terry Rudolph. Quantum coherence, time-translation symmetry, and thermodynamics. *Physical Review X*, 5(2):021001, 2015.

[6] Piotr Ćwikliński, Michał Studziński, Michał Horodecki, and Jonathan Oppenheim. Limitations on the evolution of quantum coherences: Towards fully quantum second laws of thermodynamics. *Physical review letters*, 115(21):210403, 2015.

[7] Álvaro M Alhambra, Lluis Masanes, Jonathan Oppenheim, and Christopher Perry. The second law of quantum thermodynamics as an equality. Preprint at https://arxiv.org/abs/1601.05799, 2016.

[8] Paul Skrzypczyk, Anthony J Short, and Sandu Popescu. Work extraction and thermodynamics for individual quantum systems. *Nature communications*, 5, 2014.
